# Etiologies and Outcomes in Revision Lumbar Surgery: A Single-Center Cohort Study of Patients Undergoing Two or More Revisions

**DOI:** 10.7759/cureus.99221

**Published:** 2025-12-14

**Authors:** Bharat R Dave, Mikeson Panthackel, Ajay Krishnan, Shivanand C Mayi, Ravi Ranjan Rai, Mirant B Dave, Rohan Killekar, Arjit Vashishtha, Amritesh Singh, Mahesh Sagar, Saurabh S Kulkarni, Yogenkumar Adodariya

**Affiliations:** 1 Spine Surgery, Stavya Spine Hospital and Research Institute, Ahmedabad, IND; 2 Spine Surgery, Bhavnagar Institute of Medical Science (BIMS), Bhavnagar, IND; 3 Orthopaedics, Lokmanya Tilak Municipal Medical College (LTMMC) and Sion Hospital, Mumbai, IND; 4 Orthopaedics, Geetanjali Medical College and Hospital, Udaipur, IND; 5 Orthopaedics, University College of Medical Sciences and Guru Teg Bahadur Hospital, Delhi, IND; 6 Orthopedics, Mahatma Gandhi Medical College and Research Institute, Aurangabad, IND

**Keywords:** etiology, multiple revisions, outcomes, revision, spine surgery

## Abstract

Introduction

The incidence of revision lumbar spine surgery has increased with the growing number of spinal procedures performed worldwide. Patients requiring multiple revisions represent a complex subgroup with higher complication risks and variable outcomes. This study aimed to identify the primary etiologies leading to repeated lumbar spine surgeries and to evaluate associated clinical outcomes.

Materials and methods

This single-center retrospective cohort study included 66 patients who underwent at least two revision lumbar spine surgeries (three total procedures including the index) between January 2017 and January 2022. Demographic, operative, and clinical data were extracted from hospital records. Functional outcomes were assessed using the modified Oswestry Disability Index (ODI) and Visual Analogue Scale for leg pain (VAS-LP) before and after each surgery. Statistical analysis included repeated-measures tests and Kaplan-Meier survival analysis using IBM SPSS Statistics for Windows, Version 25 (Released 2017; IBM Corp., Armonk, New York, United States).

Results

Among 66 patients undergoing at least two revision lumbar spine surgeries, infection was the predominant etiology, increasing from 7.5% at the index surgery to 63% by the second revision. Adjacent segment degeneration and implant failure accounted for 25% and 4.5% of cases, respectively. The mean interval between the index and first revision was approximately 3.6 years, which shortened to 1.9 years between the first and second revisions; infection-related cases showed the fastest recurrence (~1.3-1.5 years). Functional outcomes demonstrated significant improvement across all stages, with mean ODI and VAS-leg pain (VAS-LP) scores improving postoperatively (p < 0.001, repeated-measures analysis).

Conclusion

Infection was the leading cause of repeated lumbar spine surgeries and correlated with progressively shorter revision intervals. Despite increased surgical complexity, meaningful functional and pain improvements remained achievable. Preventing infection and ensuring careful patient selection are essential for optimizing outcomes in recurrent lumbar spine surgery.

## Introduction

The rate of revision lumbar surgery has increased over the past two decades, coinciding with a rise in the overall number of spinal procedures performed and an aging population [1,2]. However, with this rise comes a corresponding increase in the number of failed back surgeries, many of which necessitate revision interventions.

Revisions in lumbar spine surgery can be necessitated by a variety of factors and are often classified based on timing. Early surgical failures are commonly due to issues such as inadequate decompression, iatrogenic instability, or postoperative infection [3]. In contrast, late failures often involve recurrent disc herniation, adjacent segment disease, or mechanical failure of implants [4]. Revision procedures are typically more complex, technically demanding, and carry a higher risk of complications than primary surgeries. The reoperation rates, especially in decompression-only surgeries, are significant. Studies have reported 2- to 4-year reoperation rates of approximately 8-10% for such cases [5].

Various risk factors have been associated with an increased likelihood of revision spine surgeries. These include patient-related factors such as obesity, diabetes mellitus, smoking, and other comorbidities, as well as surgical variables like long fusion constructs or the type of index procedure performed. A recent meta-analysis of reoperation rates following surgical procedures for lumbar canal stenosis revealed a reoperation rate of 7.5% for fenestration and 12.7% for laminectomy [6]. Interestingly, the addition of fusion to laminectomy did not significantly increase the revision rate, though it did correlate with a higher complication profile. Understanding these risk factors is critical for both preoperative patient counseling and surgical planning.

While several studies have analyzed first revision outcomes, few have focused on patients undergoing multiple revisions, a subgroup with distinct challenges and poorer prognosis. Given the rising number of spine surgeries and the complexities involved in revision procedures, there is a pressing need for studies to find the main etiologies to guide clinical decisions. This study aims to identify the predominant etiological factors and analyze the functional outcomes (ODI, VAS) in patients undergoing at least two lumbar spine revision surgeries.

## Materials and methods

This study was designed as a single-center, retrospective, observational analysis conducted at our institution.

Patient selection

The hospital management system was utilized to identify and retrieve records of patients who had undergone at least two revision surgeries (a total of three including the index procedure) for lumbar spine pathology from January 2017 to January 2022. Ethics approval: Ethical approval was obtained from the Institutional Ethics Committee (Protocol SHRI/CS/NS/Multiple Sx/BRD/65/11.23). Consent was waived by the committee due to the retrospective design. Trial registration: CTRI/2024/01/061235.

Inclusion Criteria

Adults aged ≥18 years who underwent lumbar spine surgery involving levels T12-S1 and subsequently required two or more revision operations (a total of three procedures including the index) for degenerative, traumatic, or infective pathology between January 2017 and January 2022 were included. Surgeries performed at outside hospitals were included if operative details were available in full. Discharge summary, operative notes, and referral notes were collected from patients who underwent surgery at an outside hospital.

Exclusion Criteria

Patients with <24 months follow-up after the final revision and incomplete operative records were excluded.

Primary outcomes were (a) the distribution of revision etiologies across surgical stages and (b) the time interval between the index and first revision, and between the first and second revisions. Secondary outcomes were changes (Δ) in the modified Oswestry Disability Index (ODI) and Visual Analogue Scale for leg pain (VAS-LP). Preoperative assessments were obtained within six weeks before surgery, and postoperative follow-up scores were recorded at 12-24 months after each stage. Clinically meaningful improvement was defined as a change meeting the minimal clinically important difference (MCID): ≥12 points for ODI and ≥2 points for VAS-LP.

Preoperative clinical parameters, including Visual Analogue Scale (VAS - LP) [7] scores for leg pain and modified Oswestry Disability Index (ODI) scores [8-10], were retrieved. The modified version of the ODI which is a freely available as PDF and online calculator, has similar accuracy to the original ODI [10]. For our study, PDF was downloaded and used [9,11]. Corresponding postoperative VAS- LP scores for leg pain and ODI scores were also collected following each surgical intervention. In addition, the causes leading to revision surgeries were reviewed and categorized. Complications such as infection, implant failure, neurological deterioration, or wound-related issues were noted wherever applicable.

Etiologies (infection, ASD, implant failure) were defined using standardized clinical, radiological, and microbiological criteria and adjudicated by two fellowship-trained spine surgeons; disagreements were resolved by consensus. Infection was defined by intraoperative or radiological evidence of collection, elevated inflammatory markers, and either positive culture or purulent discharge. Tissue samples were obtained intraoperatively from deep spinal compartments using sterile instruments before irrigation. Multiple specimens were sent for aerobic and anaerobic bacterial cultures, fungal cultures, and AFB testing. Hardware explants were swabbed and submitted, although sonication was not performed. All cultures were incubated for standard durations (aerobic/anaerobic 5-7 days; fungal up to four weeks; AFB up to six weeks). Pre-operative antibiotics were withheld for ≥24 hours whenever clinically feasible to improve culture yield. Culture-negative infections were defined as cases with intraoperative purulence or abscess, radiological or laboratory evidence of infection (elevated CRP/ESR), and no alternative diagnosis. Culture-negative infections were classified based on consistent clinical and radiological findings with no alternative diagnosis. Superficial surgical-site infections were excluded; only deep or organ/space infections within the operative field were analyzed. Hematogenous infections arising post-discharge were included if confirmed radiologically and microbiologically. Implant failure includes screw pull out, breakage, rod fracture.

Surgical procedure

Revisions followed standard principles: debridement and implant retention for acute infection, hardware removal for persistent infection, and construct extension for mechanical or ASD failures. Minimally invasive Wiltse approach with tubular retractors was used for limited decompressions or short-segment fusions, while standard midline open approaches were reserved for implant removal or multilevel reconstructions.

At follow-up of two years, VAS and ODI scores were collected to find the improvement in outcome.

Statistical analysis

All data were compiled into a Microsoft Excel spreadsheet to create a master chart for statistical analysis, with both physical and digital records maintained for accuracy. Data abstraction was conducted by two independent reviewers trained in clinical data extraction. Ten percent of cases were randomly audited for duplicate entry, with discrepancies resolved by a senior spine surgeon. Adjudicators of etiology were blinded to outcome measures to minimize bias. Radiographs were anonymized by removing all personal identifiers to ensure patient confidentiality. Outcomes were analyzed on an available-case basis. Stage-specific analyses used all patients with complete ODI and VAS-LP data for that surgical stage. No statistical imputation was performed for missing patient-reported outcome measures. Denominators (n) are therefore reported separately for the index, first-revision, and second-revision cohorts. Statistical analyses were conducted using IBM SPSS Statistics for Windows, Version 25 (Released 2017; IBM Corp., Armonk, New York, United States). Change scores (Δ = postoperative − preoperative) were calculated for ODI and VAS-LP. Data normality was assessed using the Shapiro-Wilk test. As the same patients underwent multiple surgeries, a repeated-measures design was applied. Normally distributed variables were analyzed using repeated measures ANOVA, while the Friedman test was used for non-normal data. Post hoc comparisons were Bonferroni-adjusted to control multiple testing.

## Results

**Figure 1:**
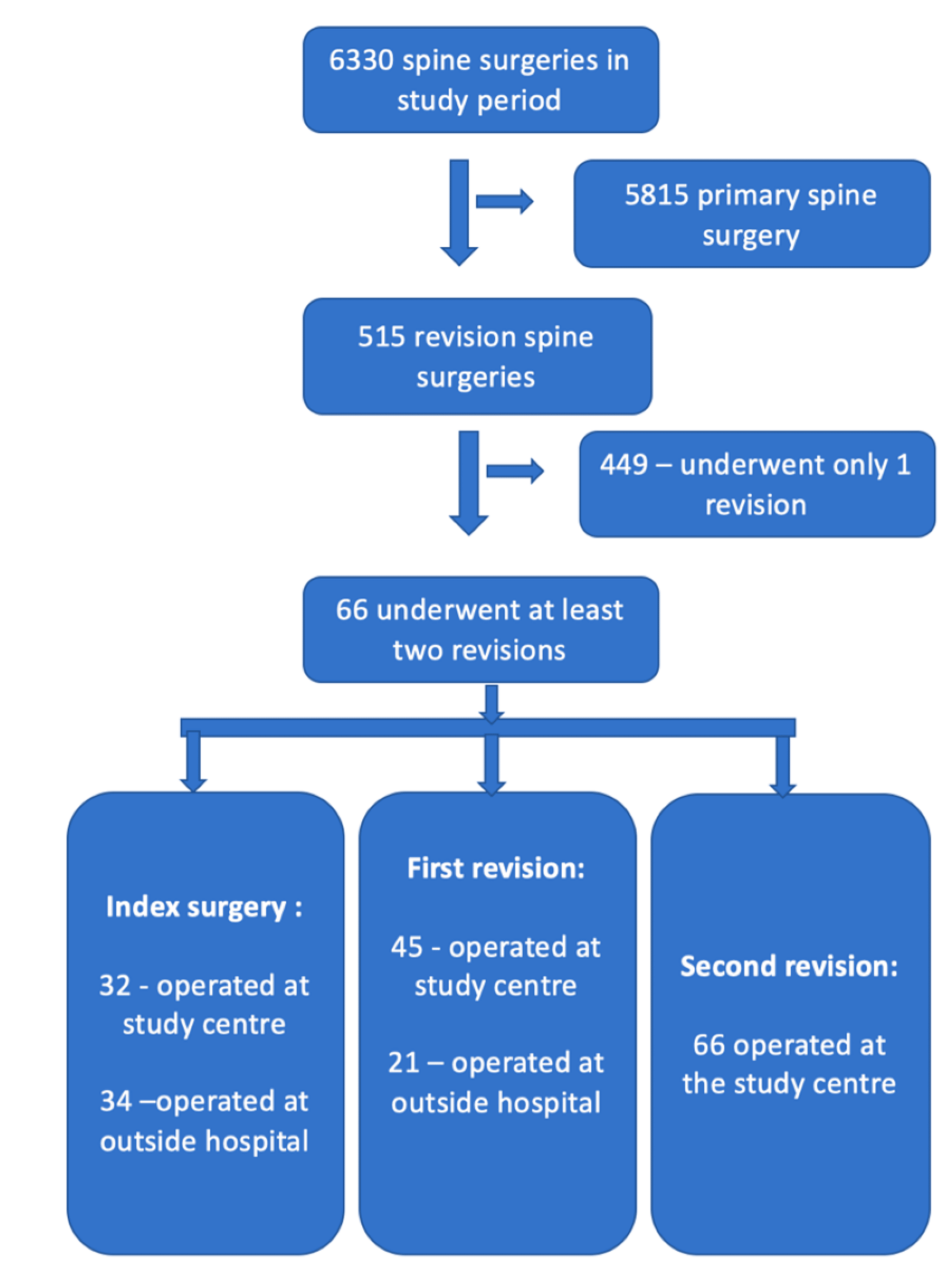
Flow diagram of cohort selection. Flow diagram summarizing identification of eligible patients and stage-wise inclusion across index, first-revision, and second-revision procedures, with differentiation between surgeries performed at the study centre and those conducted at outside hospitals.

The present study included a total of 66 patients who underwent at least two revision surgeries for lumbar spine disorders. The flowchart for the cohort selection is shown in Figure 1.

Patient data along with etiology for surgery are categorized into Table 1.

**Table 1:** Demographic data of all the cases and the frequency of etiologies in each surgery. All values are either N(%), mean ±SD, adjacent segment degeneration (ASD). Degeneration other than just adjacent levels (Degen).

Patient Characteristics and Etiology	Data	
Age	57.5±12.97	
Gender	36 male patients and 30 female patients	
Etiology for first surgery (N= 66)	Degenerative	58 (88%)
	Infectious	5 (7.5%)
	Traumatic	3 (4.5%)
Etiology for Second Surgery—First Revision (N=66)	ASD	31 (47%)
	Degen.	2 (3%)
	Infection	27 (41%)
	Implant loosening	2 (3%)
	Dural tear	1 (1.5%)
	Trauma	3 (4.5%)
Etiology for third surgery – second revision (N=66)	ASD	13 (20%)
	Degen.	3 (5%)
	Infection	42 (63%)
	Implant loosening	3 (4.5%)
	Dural tear	2 (3%)
	Others (D11 vertebroplasty retropulsion)	1 (1.5%)
	Trauma	2 (3%)

Reasons for revision were categorized separately from postoperative complications. Dural tear was included as an etiology only when it directly prompted reoperation.

Infection rates showed a progressive rise across surgical stages, increasing from 7.5% (5 of 66 cases) in the first surgery to 41% (27 of 66) during the first revision and reaching 63% (42 of 66) in second revisions, indicating a significant upward trend in infection incidence with successive procedures. A Cochran-Armitage trend test demonstrated a significant increase in infection proportion across successive surgeries (Z = 6.83, p < 0.001). Infection frequency increased progressively across revision stages. Although the Cochran-Armitage trend test suggested a significant upward pattern, this should be interpreted cautiously because repeated measures from the same patients violate independence. Overall, the most common organism encountered in infective cases was E. coli (E. coli- 21(50%), S. aureus -10(23%), E. faecalis - 2(4%), M. tuberculosis - 5(11%), all remaining cases - negative culture report 4(12%)).

Average time intervals by etiology

Based on the analysis of surgical timelines, the average interval between index surgery and the first revision was approximately 1,330 days (about 3.6 years), while the mean time between first and second revision surgeries was approximately 680 days (about 1.9 years). Notably, patients who developed infections had a shorter interval to first revision, averaging 491 days. Kaplan-Meier survival analysis demonstrated a significantly shorter time to revision in infection-related cases compared with other etiologies (log-rank χ² = 8.51, p = 0.003), as shown in Table 2.

**Table 2:** Table showing average time to revision in the entire cohort and for each category of etiology. Etiologies were divided into six main categories: degenerative, ASD, infection, implant loosening, dural tear, and traumatic causes. Two intervals were selected to find the time between revisions. The first interval is between the first surgery and the first revision, and the second interval is between the first revision and the second revision. ASD: Adjacent segment degeneration; CSF: cerebrospinal fluid

Etiology Category	Mean Time: First Surgery → First Revision (days)	Mean Time: First Revision → Second Revision (days)
Entire cohort N=66	1329.6 (3.6 years)	679.9 (1.9 years)
Degenerative	2263.5 (~6.2 years)	-
ASD	-	1399.8 (~3.8 years)
Infection	491.0 (~1.3 years)	552.6 (~1.5 years)
Implant loosening/Cage backout	1528 (~4.2 years)	137.2 (~4.5 months)
Dural tear/CSF leak	30.0 (~1 month)	210.0 (~7 months)
Traumatic	361.0 (~1.0 year)	922.5 (~2.5 years)

**Figure 2:**
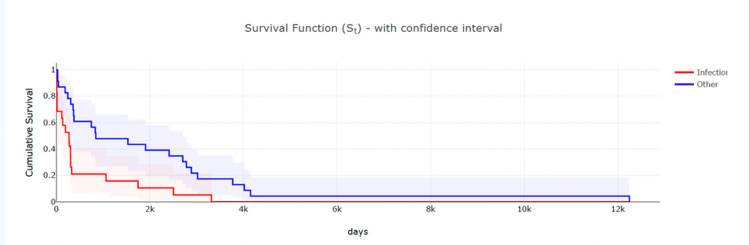
Kaplan–Meier curve for time to revision stratified by etiology. This Kaplan–Meier survival curve displays the time from the preceding surgery to the next revision, stratified by infection-related versus non-infection etiologies. Patients with infection (red curve) demonstrated significantly shorter survival intervals before requiring another revision compared with patients revised for other causes (blue curve). Shaded bands represent 95% confidence intervals. The curves illustrate the accelerated failure pattern in infection-related cases, consistent with the cohort’s overall trend toward progressively shorter intervals between successive surgeries.

The Kaplan-Meier figure is shown in Figure 2.

Among the 66 patients who underwent a second revision at our institute, only a subset had also undergone their first revision at the same center, and an even smaller number had their index surgery performed here. Consequently, complete outcome data (ODI and VAS-LP) were available only for surgeries performed at our institution, whereas for cases operated elsewhere, only operative details and etiology data could be retrieved. Therefore, the sample size (n) varied across surgical stages depending on the availability of validated pre- and postoperative patient-reported outcomes. The number of cases with data on PROMS available was index n = 32, first revision n = 45, and second revision n = 66. Stage-wise analyses were conducted on an available-case basis, and denominators are reported separately in the results tables. No imputation was performed for missing outcome data.

With respect to surgical complications after the revision procedure (within 90 days), the most common adverse event encountered was dural tear, observed in six surgeries. Other complications included foot drop and wound-related issues necessitating re-intervention. While no uniform pattern was observed in terms of specific procedures preceding dural tears, a number of these cases had undergone complex or multi-level interventions. All dural defects were repaired primarily using non-absorbable 4-0 braided silk sutures. When primary closure was not possible due to friable tissue or narrow exposure, the tear was reinforced with a collagen onlay graft and fibrin sealant. A Valsalva maneuver was performed after repair to confirm the absence of leakage. Postoperatively, patients were maintained on flat bed rest for 24-48 hours with gradual mobilization thereafter.

Statistical analysis demonstrated a significant difference in functional improvement across surgical stages for the modified ODI (p < 0.001). A similarly significant difference was observed in pain reduction measured by the VAS-LP (p < 0.001). Improvement was noted in 95% of index, 78% of first revision, and 69% of second revision cases, indicating effective symptom relief despite a progressive decline in response rates with successive surgeries.

Patients undergoing second-revision procedures exhibited the highest baseline disability (mean preoperative ODI = 76.1 ± 7.3) compared with first-revision (58.6 ± 8.9) and index surgeries (43.9 ± 12.3). Despite this, all groups demonstrated significant postoperative improvement.

VAS-LP scores showed substantial pain reduction across all stages-index (Δ = 4.69 ± 1.1), first revision (Δ = 4.35 ± 1.3), and second revision (Δ = 3.74 ± 0.9). Data are shown in Table 3.

**Table 3:** Comparison of functional (ODI) and pain (VAS-LP) outcomes after index and revision spine surgeries This table summarizes the mean preoperative and postoperative modified Oswestry Disability Index (ODI) and Visual Analogue Scale- Leg Pain (VAS- LP) scores across three surgical stages: index, first revision(firstrev), and second revision (secrev). Paired t-tests were used to assess within-stage improvements. All stages demonstrated statistically significant postoperative improvement (p < 0.001) in both ODI and VAS-LP scores.

Outcome	Stage	n	Mean Pre	Mean Post	Mean Improvement (Pre-Post)	Paired t	p-value
ODI	index	32	43.94	25.69	18.25	6.439	<0.001
ODI	firstrev	45	58.59	30.09	28.5	18.883	<0.001
ODI	secrev	66	76.06	42.18	33.88	18.389	<0.001
VAS	index	32	6.88	2.19	4.69	13.896	<0.001
VAS	firstrev	45	7.53	3.18	4.35	14.516	<0.001
VAS	secrev	66	7.17	3.42	3.74	11.924	<0.001

Analysis shows that although patients undergoing revision surgeries present with greater baseline and postoperative disability, they still experience proportionally larger functional gains, while the degree of pain relief remains uniform across all surgical stages.

Inter-stage comparison revealed a progressive increase in functional improvement with successive surgeries. The mean ODI change was significantly greater in first and second revisions compared to index surgeries (p < 0.01), reflecting higher baseline disability and larger absolute recovery potential. However, the difference between first and second revisions was not statistically significant. In contrast, pain relief measured by VAS-LP showed no significant variation across stages (p = 0.14), suggesting that while functional restoration diminishes with repeated procedures, effective pain reduction remains achievable even in complex revision cases. These findings emphasize the therapeutic value of revision spine surgery despite diminishing overall functional gains.

To account for the repeated nature of outcome measurements across surgical stages within the same patients, a repeated-measures ANOVA was performed for both ODI and VAS-LP scores. Only cases with complete pre- and postoperative data for a given stage were included in each analysis, yielding stage-specific denominators as reported earlier (index n = 32, first revision n = 45, second revision n = 66). Both models demonstrated a significant main effect of time, indicating consistent postoperative improvement across all stages. The stage effect and the time-by-stage interaction were used to evaluate whether baseline severity and the magnitude of improvement differed between index, first-revision, and second-revision surgeries. The detailed ANOVA results for ODI and VAS-LP are presented in Tables 4, 5.

**Table 4:** Repeated-measures ANOVA for change in modified Oswestry Disability Index (ODI) across surgical stages This table shows the repeated-measures ANOVA comparing preoperative and postoperative ODI scores across index, first-revision, and second-revision surgeries. The analysis demonstrated a significant main effect of timepoint, confirming postoperative improvement in disability. A significant main effect of surgical stage indicated differences in disability severity between stages, and the significant timepoint × stage interaction showed that the degree of ODI improvement varied across surgeries, with larger gains observed in revision stages.

Effect	F	df1	df2	p	η²p
Surgery	88.15	2	62	<0.001	0.74
Timepoint	371.06	1	31	<0.001	0.923
Surgery × Timepoint	6.64	2	62	0.002	0.176

**Table 5:** Repeated-measures ANOVA for change in visual analogue scale for leg pain (VAS-LP) across surgical stages This table presents the repeated-measures ANOVA evaluating changes in VAS-LP across the three surgical stages. A significant main effect of timepoint confirmed consistent postoperative reduction in leg pain. The main effect of stage indicated differences in baseline pain levels between surgeries, while the significant timepoint × stage interaction showed that the magnitude of VAS-LP improvement differed modestly across stages, though pain relief was evident following all procedures.

Effect	F	df1	df2	p	η²p
Surgery	5.07	2	62	0.009	0.141
Timepoint	285.35	1	31	<0.001	0.902
Surgery × Timepoint	3.38	2	62	0.041	0.098

Clinically meaningful improvement was achieved by a majority of patients across all surgical stages. For the ODI, where the MCID was defined as a reduction of ≥12 points, 68.8% of patients (95% CI, 51.4%-82.0%) met this threshold after their index surgery. This proportion was higher following revision procedures, with 93.8% (95% CI, 79.9%-98.3%) achieving MCID after the first revision and 87.5% (95% CI, 71.9%-95.0%) after the second revision. For the VAS-LP, defined by an MCID of ≥2 points, 93.8% of patients (95% CI, 79.9%-98.3%) experienced a clinically important reduction after index surgery. Similarly, 96.9% of patients (95% CI, 84.3%-99.4%) met the VAS-LP MCID after their first revision, and 78.1% (95% CI, 61.2%-89.0%) did so after their second revision.

## Discussion

Infection emerged as a leading cause, 42 (63%) of multiple lumbar spine revision surgeries (second revision surgery) in this cohort (p-value <0.001). Infections are a well-established cause of multiple surgeries in the lumbar spine. Postoperative spinal infections frequently necessitate additional surgeries due to persistent or recurrent infection, wound complications, pseudoarthrosis, and hardware involvement. For example, in a large retrospective cohort, infection accounted for 28% of all lumbar spine reoperations, making it the most common cause for revision, particularly within the first month after the primary procedure ​[12,13]. In one study, all infected cases required at least one reoperation for surgical debridement and sometimes hardware removal. Effective prevention, early diagnosis, and aggressive management of spinal infections are therefore critical to minimizing the need for repeated surgeries and reducing associated morbidity [14].

Not only is infection the major cause of multiple revision surgeries, but the risk of developing an infection also increases with each successive revision compared to primary spine surgery. Furthermore, the risk of infection increases with the number of fused levels in cases of instrumentation, suggesting the need for preoperative risk stratification and vigilant surveillance for infection in revision cases [15,16]. In our study, all de novo infections after index surgeries occurred in cases where fusion was the primary procedure, except for two cases in which discectomy was performed. None of the de novo cases had laminectomy as their first surgery.

The average time to reoperation for spinal infection varies across studies, but the majority occur within 30 days after the initial surgery. According to a large retrospective cohort study, 71% of patients who underwent reoperation due to infection did so within 30 days of their initial spine procedure. Other studies report mean times to reoperation ranging between 11 and 35 days, with most infections presenting acutely in the first month, although some late infections can occur several months or even years after the original surgery. Early identification and management of infection are therefore critical to reducing morbidity and improving outcomes in spine surgery patients.​ In our study, the mean time before revision was notably shorter for infection cases (491 days) compared to degenerative cases (1330 days), indicating a faster progression to failure in infection-related revisions. Because this study includes only patients who survived through two or more revision procedures, the cohort is biased toward individuals with delayed or late surgical failures. As a result, the reoperation intervals in our dataset are substantially longer than the early (≤30-day) reoperations frequently described in the infection literature. We hypothesize that primary surgeons’ preference for extended antibiotic management over early reoperation in infection cases leads to prolonged time-to-revision surgery in some infection cases. Additionally, many of these patients had undergone their primary surgeries outside our institution [17,18].

The average intervals between revisions in our study, approximately 3.6 years from the index procedure to the first revision and 1.9 years to the second, suggest an accelerating pattern of surgical failure in recurrent cases. This could be due to the increasingly complex and debilitating nature of failed back surgery. The importance of the time to intervene in revision cases has been studied in a prospective study of 98 FBSS patients where they found that those who had shorter symptom duration before revision surgery are associated with higher postoperative success, likely due to less chronic pain and neural adaptation when surgery is performed sooner after recurrence [19].

Other than infections, implant failure and hardware failure were also important complications contributing to revision spinal surgery. In their study of non-infective causes, Passias et al. reported a revision rate of 16.5% due to non-infective factors [20]. In our study, 16 (25%) were attributable to adjacent segment degeneration, 3(4.5%) were due to implant failure, 2(3%) were related to fractures at adjacent levels, 2 (3%) were due to pseudomeningocele causing symptoms, and one (1.5%) was due to D11 retropulsion in a case of postop vertebroplasty. Among the implant failure cases, two had long fixations D10- L5, which had to be extended to the pelvis. One of them again had to be revised due to implant failure.

The rates of complications and length of hospital stay are higher in revision spine surgery as compared to primary spine surgery. Common complications encountered in these cases include dural tears and infections [15,21]. In our study, dural injury was the most frequently observed surgical complication, occurring in six cases. In primary spine surgeries, the risk of dural tears was 5.8%; however, this risk increases in patients undergoing revision surgery [22]. The risk of dural tears increases in revision surgery due to altered anatomical planes and dense scar tissue. Interestingly, a subset of these dural tears was associated with infection, suggesting a potential synergistic relationship in which infection-related tissue degradation may predispose to dural compromise. Four of the seven cases of dural tears in our study were observed in patients undergoing multilevel and complex procedures.

In all the revisions, the mean ODI and VAS-LP scores improved compared to pre-op. Clinically, the high proportion of patients exceeding the MCID thresholds for both ODI and VAS-LP indicates that the observed improvements were not only statistically significant but also meaningful from the patient’s perspective. These findings support the role of both index and revision lumbar procedures in delivering tangible functional and pain benefits, even in a cohort with progressively worsening preoperative disability.

Though revision spine surgery in general can yield good results when performed in appropriate cases, outcomes and patient satisfaction are significantly lower in revision surgery as compared to primary spine surgery [23,24]. Revision surgery for pseudoarthrosis in particular has demonstrated poorer outcomes than revision for instability [21,24]. Furthermore, studies comparing revision for pseudoarthrosis to revision for adjacent segment disease have shown poorer outcomes with revision for pseudoarthrosis [25]. Rates of 70% are similarly seen in studies on outcomes after revision surgery [19].

Nevertheless, this study has several limitations that warrant consideration. The retrospective nature of the analysis may introduce biases related to patient selection and data collection. Furthermore, the small sample size limits the statistical power and generalizability of the findings to a broader population of spinal surgery patients. Additionally, the study relied predominantly on clinical follow-up data. Importantly, cases of infection were frequently referred to specialized care, which may have resulted in incomplete data for these cases and an underestimation of their true prevalence and complications. Future prospective, large-scale studies with more exhaustive follow-up and standardized data collection methods are required to validate these findings and provide more robust conclusions.

Additional limitations include incomplete PROMs at the index and first-revision stages, resulting in an available-case analysis; inherent survivorship bias because only patients who progressed to multiple revisions were captured; a non-standardized infection workup (no sonication and variable culture techniques), which may affect organism detection; absence of multivariable adjustment for confounders; and the single-center referral nature of the cohort, which limits generalizability.

## Conclusions

This retrospective study provides valuable insight into the patterns, causes, and outcomes of multiple revision lumbar spine surgeries in patients with degenerative disorders. Infection was identified as the leading cause of repeated interventions (63%) underscoring its significant role in spinal surgery failure. Furthermore, the average interval between successive surgeries progressively shortened. Although revision surgery provided significant pain relief, the base line and postoperative scores were on the higher side with each subsequent procedure. These findings underscore the necessity for careful patient selection, rigorous perioperative protocols, and tailored strategies to reduce revision rates.
